# Initial Metabolic Step of a Novel Ethanolamine Utilization Pathway and Its Regulation in *Streptomyces coelicolor* M145

**DOI:** 10.1128/mBio.00326-19

**Published:** 2019-05-21

**Authors:** Sergii Krysenko, Arne Matthews, Nicole Okoniewski, Andreas Kulik, Melis G. Girbas, Olga Tsypik, Christian Stephan Meyners, Felix Hausch, Wolfgang Wohlleben, Agnieszka Bera

**Affiliations:** a Department of Microbiology and Biotechnology, Interfaculty Institute of Microbiology and Infection Medicine Tübingen (IMIT), University of Tübingen, Tübingen, Germany; b Center for Biological Systems Analysis, University of Freiburg, Freiburg, Germany; c Celerion Switzerland AG, Fehraltdorf, Switzerland; d Institute for Molecular Biosciences, University of Frankfurt, Frankfurt am Main, Germany; e Department of Pharmaceutical Biology and Biotechnology, Institute of Pharmaceutical Sciences, Albert-Ludwigs-University of Freiburg, University of Freiburg, Freiburg, Germany; f Institute of Organic Chemistry and Biochemistry, Technical University Darmstadt, Darmstadt, Germany; Max Planck Institute for Terrestrial Microbiology

**Keywords:** *Streptomyces*, ethanolamine utilization, gamma-glutamylation, nitrogen metabolism

## Abstract

Streptomyces coelicolor is a Gram-positive soil bacterium with a high metabolic and adaptive potential that is able to utilize a variety of nitrogen sources. However, little is known about the utilization of the alternative nitrogen source ethanolamine. Our study revealed that S. coelicolor can utilize ethanolamine as a sole nitrogen or carbon (*N*/*C*) source, although it grows poorly on this nitrogen source due to the absence of a specific ethanolamine permease. Heterologous expression of a putative ethanolamine permease (SPRI_5940) from Streptomycespristinaespiralis positively influenced the biomass accumulation of the overexpression strain grown in defined medium with ethanolamine. In this study, we demonstrated that a glutamine synthetase-like protein, GlnA4 (SCO1613), is involved in the initial metabolic step of a novel ethanolamine utilization pathway in S. coelicolor M145. GlnA4 acts as a gamma-glutamylethanolamide synthetase. Transcriptional analysis revealed that expression of *glnA4* was induced by ethanolamine and repressed in the presence of ammonium. Regulation of *glnA4* is governed by the transcriptional repressor EpuRI (SCO1614). The *ΔglnA4* mutant strain was unable to grow on defined liquid Evans medium supplemented with ethanolamine. High-performance liquid chromatography (HPLC) analysis demonstrated that strain *ΔglnA4* is unable to utilize ethanolamine. GlnA4-catalyzed glutamylation of ethanolamine was confirmed in an enzymatic *in vitro* assay, and the GlnA4 reaction product, gamma-glutamylethanolamide, was detected by HPLC/electrospray ionization-mass spectrometry (HPLC/ESI-MS). In this work, the first step of ethanolamine utilization in S. coelicolor M145 was elucidated, and a putative ethanolamine utilization pathway was deduced based on the sequence similarity and genomic localization of homologous genes.

## Abstract (precis)

Until now, knowledge of the utilization of ethanolamine in *Streptomyces* was limited. Our work represents the first attempt to reveal a novel ethanolamine utilization pathway in the actinobacterial model organism S. coelicolor through the characterization of the key enzyme gamma-glutamylethanolamide synthetase GlnA4, which is absolutely required for growth in the presence of ethanolamine. The novel ethanolamine utilization pathway is dissimilar to the currently known ethanolamine utilization pathway, which occurs in metabolome. The novel ethanolamine utilization pathway does not result in the production of toxic by-products (such as acetaldehyde); thus, it is not encapsulated. We believe that this contribution is a milestone in understanding the ecology of *Streptomyces* and the utilization of alternative nitrogen sources. Our report provides new insight into bacterial primary metabolism, which remains complex and partially unexplored.

## INTRODUCTION

Ethanolamine belongs to a class of aliphatic amino alcohols, and it represents both a primary amine and a primary alcohol. Ethanolamine is widely distributed in mammalian and bacterial cell membranes (1, 2), where it is a constituent of phospholipids such as phosphatidylethanolamine. Phosphatidylethanolamine composes 25% to 45% of all phospholipids in a cell and is an important substrate and precursor in several biological pathways (2). As a principal phospholipid in bacteria, phosphatidylethanolamine plays a major role in scattering the negative charge caused by the presence of anionic membrane phospholipids. It enables the proper functioning of transporters and acts as a chaperone to help membrane proteins fold correctly (3, 4). In most bacteria, phosphatidylethanolamine is synthetized via phosphatidylserine decarboxylation (5). Ethanolamine can serve as a precursor of phosphatidylethanolamine in a pathway that includes phosphatidylserine (6) and ethanolamine phosphate (7, 8).

Ethanolamine could have originally formed from NH_3_/NH_4_^+^ in hydrothermal environments on early Earth (9). Since ethanolamine is a common constituent of phospholipids, its omnipresence in all life kingdoms is unsurprising. Ethanolamine can be released into the soil during the decomposition of the organic matter derived from dead plants, animals, fungi, and microorganisms, or it can arrive into the soil as a component of fertilizers or as an unintentionally introduced pollutant (9, 10). Ethanolamine has an antimicrobial effect due to its surface-active properties, and in high concentrations it can effectively inhibit growth of some microorganisms in the soil (9). However, the antimicrobial effect is more significant to autotrophic nitrifying bacteria than to heterotrophic bacteria (11), which seem benefit from this alternative *C*-/*N*-source, ensuring their survival under fluctuating nutritional conditions in the soil.

A variety of bacteria, including Gram-negative bacteria such as *Salmonella*, *Escherichia* (12), *Klebsiella* (12), *Erwinia*, *Flavobacterium*, *Achromobacter*, *Pseudomonas* (13), and *Vibrio* (14) and Gram-positive bacteria such as *Enterococcus* (15), *Arthrobacter*, *Corynebacterium* (12), *Clostridium*, *Listeria* (16, 17), *Streptococcus* (15), and *Mycobacterium* (6–8), can utilize ethanolamine as a sole source of carbon and/or nitrogen (18). Bacteria are not able to synthesize ethanolamine *de novo*; however, extracellular ethanolamine can enter bacterial cells through diffusion or carrier-mediated transport (19). Ethanolamine catabolic genes are located together in an ethanolamine utilization operon (*eut*). Phylogenetic analysis of almost 100 fully sequenced bacterial genomes revealed the presence of differently organized *eut* operons (18). Some *Actinobacteria* and *Proteobacteria* species have short *eut* operons containing only the following three genes: *eutB* and *eutC* (*eutBC*; encoding a central enzyme of the ethanolamine utilization process—ethanolamine ammonia lyase) and *eat* (encoding an ethanolamine transporter). Some *Proteobacteria* may also contain *eutR* (encoding a transcriptional regulator EutR of the *eut* operon). Members of Enterobacteriaceae, which includes Salmonella enterica serovar Typhimurium and Escherichiacoli, as well as members of *Firmicutes*, possess considerably different and long *eut* operons (18, 20, 21).

Ethanolamine utilization has been extensively studied in the model organism *S.* Typhimurium for over 40 years (22)*. S.* Typhimurium possesses a *eut* operon containing 17 genes encoding proteins involved in ethanolamine transport, metabolism, and regulation. All essential enzymes involved in ethanolamine utilization in this bacterium are located in a metabolosome—a multiprotein complex (carboxysome-like complex). Utilization of ethanolamine involves splitting this compound with an ethanolamine ammonia lyase (EutBC) into ammonia and acetaldehyde, which is further converted into acetyl-coenzyme A (acetyl-CoA) (23). The resulting end products, ammonium and acetyl-CoA, serve as a cellular supply of the easily assimilable *N* and *C* sources, respectively (24). Encapsulation of this metabolic pathway prevents loss of the volatile intermediate acetaldehyde and protects the cell from its potential toxicity (25, 26). Although ethanolamine utilization in a metabolosome has been broadly studied in evolutionarily diverse bacteria, the ethanolamine utilization pathway is not universal for all microorganisms.

Hardly anything is known about ethanolamine utilization and its regulation in the genus *Streptomyces*. The model organism Streptomyces coelicolor is a filamentous Gram-positive, nonmotile, obligate aerobic soil-dwelling bacterium with high G-C content and belongs to the genus *Streptomyces*, phylum *Actinobacteria*. Soil, a natural habitat of S. coelicolor, exhibits highly diverse levels of nutrient availability ranging from nutrient-poor to nutrient-rich conditions, depending on the soil type and seasonal changes. This bacterium can assimilate nitrogen from a variety of nitrogen sources, such as ammonium, nitrate/nitrite, amino acids, peptides, urea, and amino sugars. The utilization of alternative nitrogen sources reflects the elaborative survival strategy of *Streptomycetes* spp. in their natural environment. The capacity of S. coelicolor to utilize alternative nitrogen sources such as polyamines (putrescine, cadaverine, and spermidine) has been recently demonstrated (27).

The high metabolic potential of *Streptomycetes* ensures the adaptation of these bacteria to a wide variety of ecological niches and successful competition with other microorganisms for space and resources in their habitat. Distinct ecological niches occupied by *Streptomyces* exerted specific evolutionary pressure on glutamine synthetase (GS) genes (*glnA*), resulting in the evolution of diverse but thus far uncharacterized *glnA*-like genes (27, 28). Indeed, Streptomyces coelicolor A3(2) harbors two genes, *glnA* (*SCO2198*) and *glnII* (*SCO2210*), encoding glutamine synthetases GSI and GSII, whose function and regulation have been extensively studied (29–31), as well as three other genes, *glnA2* (*SCO2241*), *glnA3* (*SCO6962*), and *glnA4* (*SCO1613*), annotated as encoding putative GS-like enzymes (32). As reported by Krysenko et al. (27), the *glnA*-like genes may encode proteins annotated as glutamine synthetase-like (GS-like) enzymes that may represent as-yet-unrecognized enzymes that catalyze the gamma-glutamylation of different substrates. In this work, we demonstrated the involvement of the GlnA-like enzyme GlnA4, a gamma-glutamylethanolamide synthetase, in a novel pathway for ethanolamine degradation in S. coelicolor M145.

## RESULTS

### Ethanolamine is not an optimal nitrogen source for S. coelicolor M145.

**FIG 1:**
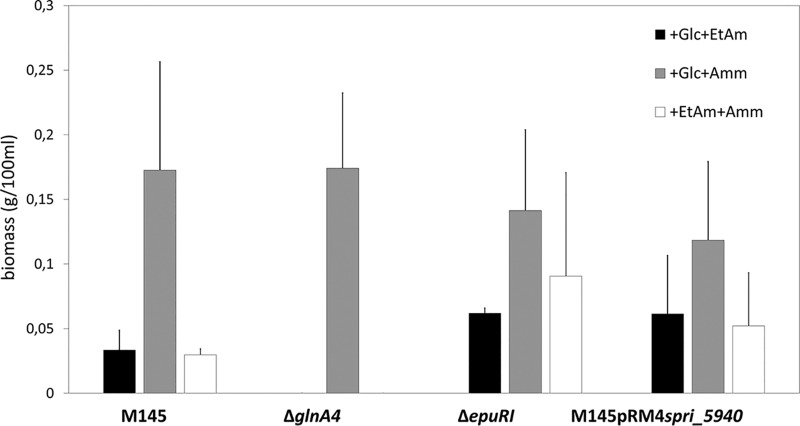
Effect of ethanolamine (25 mM) as a sole nitrogen or carbon source on the biomass accumulation of the M145 parental strain, *glnA4* mutant, *epuRI* mutant, and putative ethanolamine permease overexpression strain M145pRM4*spri_5940* in defined Evans medium after 7 days of incubation at 30°C. Error bars indicate standard errors of results from *n* = 3 biological replicates.

In this study, the ability of Streptomyces coelicolor M145 to grow on ethanolamine and utilize it as a *C* or *N* source was assessed. For this purpose, S. coelicolor M145 was grown in defined medium (Evans), supplemented either with ethanolamine (as the sole *N* source) and glucose (*C* source) or with ethanolamine (as the only *C* source) and ammonium (*N* source). As a control, S. coelicolor M145 was incubated in defined medium (Evans) supplemented with ammonium (as the sole *N* source) and glucose (*C* source). The concentration of ethanolamine and ammonium was 25 mM. The biomass accumulation was determined after 7 days of incubation at 30°C and 180 rpm. S. coelicolor M145 demonstrated very low biomass accumulation in the cultures containing ethanolamine either as the *C* source or *N* source. The biomass accumulation in the control culture with ammonium as the sole *N* source was 5 times higher than that in the culture supplemented with ethanolamine as the sole *N* source (Fig. 1). This result indicates that ethanolamine is definitely not the preferred nutrient source for S. coelicolor M145. The reason for the low biomass accumulation during growth on ethanolamine might have been either the incapability of efficient ethanolamine uptake or the impaired ethanolamine utilization under the tested conditions.

### The conserved ethanolamine permease gene is absent from the S. coelicolor genome.

**FIG 2:**
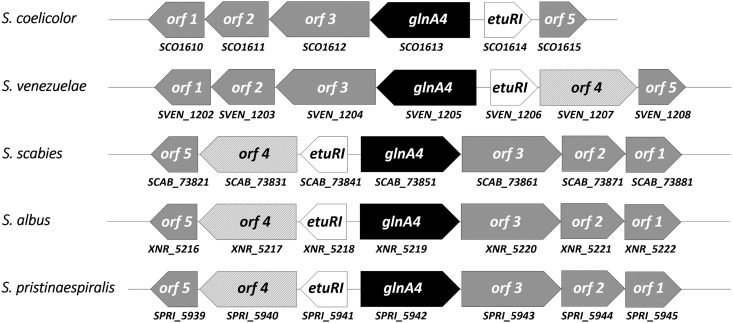
Identification and organization of the novel ethanolamine utilization gene cluster in representative *Streptomyces* sp. genomes (S. coelicolor, S. venezuelae, S. scabies, S. albus, and S. pristinaespiralis). The proposed gene product functions are as follows: *orf1*, membrane protein; *orf2*, short-chain dehydrogenase; *orf3*, aldehyde dehydrogenase; *glnA4*, gamma-glutamylethanolamide synthetase; *etuRI*, ethanolamine utilization pathway regulator; *orf4*, putative ethanolamine transporter; *orf5*, glutamine amidotransferase.

To search for the presence of an ethanolamine uptake gene(s) in the S. coelicolor M145 genome-derived proteome, amino acid sequences of the previously extensively characterized ethanolamine uptake proteins EutH and Eat from E. coli and *S.* Typhimurium were used as targets for the BLASTp analysis. No strong homologs for ethanolamine permease EutH and Eat were identified in S. coelicolor M145 or other *Streptomyces* spp. However, independent screening of several annotated *Streptomyces* genomes revealed genes encoding proteins annotated as ethanolamine/amino acid permeases with no similarity to EutH and Eat. Interestingly, some *Streptomyces* spp. contain a putative ethanolamine/amino acid permease gene in their genomes (SVEN1207 in Streptomyces venezuelae, SPRI_5940 in S. pristinaespiralis, SGR_6475 in S. griseus, *XNR_5217* in S. albus, and *SCAB_73831* in S. scabies). The putative ethanolamine/amino acid permease gene is located in a thus-far-uncharacterized gene cluster that is conserved to a considerable degree across *Streptomyces* genomes. Although the gene cluster is present in the S. coelicolor genome (*SCO1610* to *SCO1615*), the gene annotated as encoding a putative ethanolamine/amino acid permease is absent from the gene cluster (Fig. 2). Comparative analysis of the putative ethanolamine permease homologs from 18 *Streptomyces* spp. by BLASTp revealed a high level of amino acid similarity (83% to 98%). Consequently, if the genome of S. coelicolor M145 contained this homolog, it would have revealed high similarity. However, screening for the putative ethanolamine permease gene in S. coelicolor M145 allowed *in silico* identification of two putative ethanolamine/amino acid permease genes, *SCO6014* and *SCO5977*, with rather low similarity to the putative ethanolamine permease target from other *Streptomycetes* spp. (43% to 45%). To verify whether the expression of *SCO6014* and *SCO5977* might be influenced by ethanolamine, their expression patterns were analyzed in the presence of ethanolamine and ammonium (control) as the sole *N* source. Transcriptional analysis of *SCO6014* and *SCO5977* revealed strong expression of these genes in the presence of ammonium, whereas no expression was observed in the presence of ethanolamine under the tested conditions (see Fig. S1 in the supplemental material). These analyses led us to assume that the S. coelicolor M145 genome does not contain any *eutH* or *eat* homologs or the putative ethanolamine permease gene (found in other *Streptomyces*). S. coelicolor M145 seems to be ethanolamine permease deficient; however, small amounts of ethanolamine could possibly enter the cell by diffusion, consistent with our observation of slow growth on ethanolamine.

10.1128/mBio.00326-19.1 FIG S1 Transcriptional analysis of *SCO6014* and *SCO5977.* Reverse transcriptase PCR was performed with *SCO6014*, *SCO5977*, and *hrdB* (control) from S. coelicolor M145 cultivated in defined Evans medium with ammonium chloride (25 mM) or ethanolamine (25 mM). Expression of *SCO6014* and *SCO5977* was not induced by ethanolamine or by ammonium. Download FIG S1, TIF file, 0.1 MB. Copyright © 2019 Krysenko et al. 2019 Krysenko et al. This content is distributed under the terms of the Creative Commons Attribution 4.0 International license.

### Heterologous expression of the putative ethanolamine permease gene *spri_5940* from S. pristinaespiralis promoted biomass accumulation during growth on ethanolamine.

Our analysis showed that ethanolamine is not a preferred *N*/*C* source and that its presence might have been a consequence of the absence of an ethanolamine permease in S. coelicolor M145. To verify whether the putative ethanolamine permease gene from S. pristinaespiralis *spri_5940* is functional and whether its activity might restore the transport of ethanolamine and improve the growth of S. coelicolor M145 on ethanolamine, heterologous expression analysis was performed. For this purpose, the putative ethanolamine permease gene *spri_5940*, located on the pRM4 plasmid under the control of the constitutively expressed promoter P*_emrE_*, was transferred into S. coelicolor M145. As a negative control, S. coelicolor M145 with pRM4 without an insertion was used. Transcriptional analysis of *spri_5940* confirmed its constitutive expression in S. coelicolor M145 pRM4*spri_5940* independently of the nitrogen source used (data not shown).

The growth of the S. coelicolor M145 pRM4*spri_5940* strain was monitored in defined Evans medium supplemented with ethanolamine (25 mM) as the sole nitrogen source after 7 days of incubation at 30°C. The heterologous expression of *spri_5940* in S. coelicolor M145 almost doubled the biomass accumulation during growth on ethanolamine as a *N*/*C* source (Fig. 1). Our results show that this gene might encode an ethanolamine permease that is needed for effective ethanolamine uptake in S. coelicolor.

### The deletion of *glnA4* caused a growth defect in the presence of ethanolamine.

**FIG 3:**
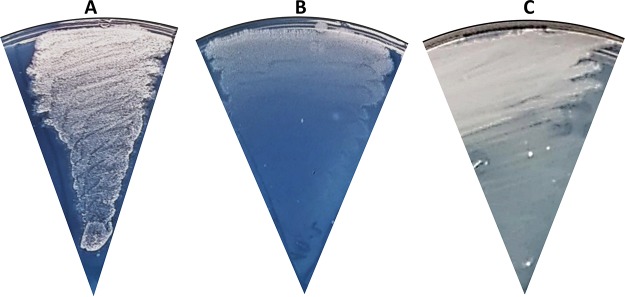
Physiological role of the *glnA4* gene product in S. coelicolor M145 cells grown in the presence of ethanolamine. Phenotypic analysis of S. coelicolor M145 (A), the Δ*glnA4* mutant (B), and the complemented mutant Δ*glnA4*pRM4*glnA4* (C) was performed on defined Evans medium with ethanolamine hydrochloride (25 mM) (A) as the sole nitrogen source. Deletion of the *glnA4* gene resulted in compromised growth on ethanolamine, whereas complementation with pRMglnA4 under the control of its native promoter restored growth on ethanolamine.

A close inspection of the conserved genomic region comprising *SCO1610* to *SCO1615* in the S. coelicolor M145 genome revealed a glutamine synthetase-like gene, *glnA4* (*SCO1613*) (Fig. 2). Recently, we showed that this gene encodes neither a glutamine synthetase nor a gamma-glutamylpolyamine synthetase (27); nevertheless, the function of GlnA4 remained unclear. Since the *glnA4* gene is most closely located to a putative ethanolamine permease gene in *Streptomycetes* genomes, we concluded that it might possibly encode a protein involved in the ethanolamine utilization pathway. As previously reported by Krysenko et al. (27), the *glnA4* mutant was able to grow on defined Evans agar supplemented with ammonium chloride, sodium nitrate, l-glutamine, monosodium l-glutamate, and polyamines (putrescine, cadaverine, and spermidine) as the sole nitrogen source. However, growth of the *glnA4* mutant had never been tested in the presence of ethanolamine. In the current study, the growth of the *glnA4* mutant was monitored after 3 to 12 days of incubation at 30°C on defined Evans agar supplemented with 25 mM ethanolamine hydrochloride. As a control, the parental strain was grown under the same conditions. Growth of S. coelicolor M145 on an ethanolamine plate resulted in delayed mycelium development, the absence of spore formation, and slight actinorhodin production, visible as a blue pigment in the agar (Fig. 3). The phenotypic analysis of the *glnA4* mutant revealed strongly delayed growth, confirming our hypothesis that GlnA4 might be involved in ethanolamine utilization. The complementation of the *glnA4* mutant with the *glnA4* gene under the control of its native promoter restored growth on an ethanolamine plate (Fig. 3).

**FIG 4:**
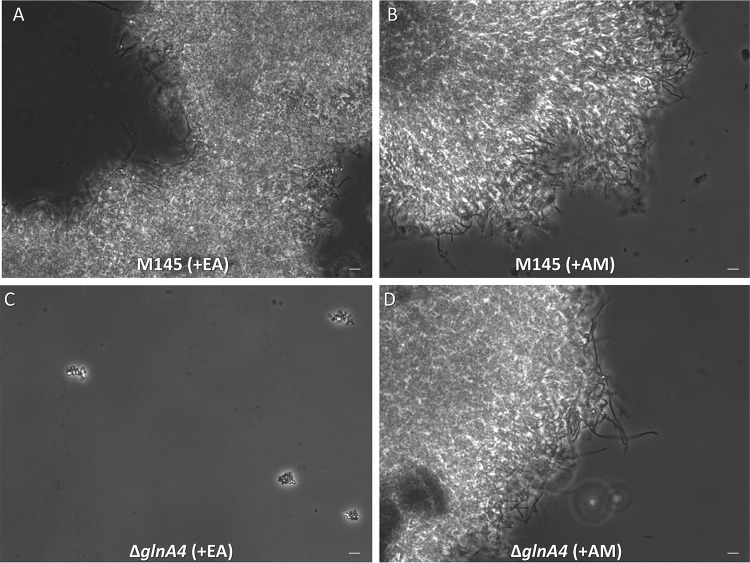
Effect of *glnA4* deletion on cell morphology in the presence of ethanolamine. The S. coelicolor M145 parental strain (A and B) and *ΔglnA4* mutant (C and D) were cultivated in defined Evans medium with ethanolamine hydrochloride (EA; 25 mM) (left panels) or ammonium chloride (AM; 25 mM) (right panels). Phase-contrast microscopic pictures of the M145 parental strain and ΔglnA4 mutant mycelium were taken (under ×400 magnification) after 96 h of growth. Bar = 5 μm.

To quantify biomass accumulation and observe cell morphology, the *glnA4* mutant and the parental strain were also grown in liquid defined Evans medium supplemented either with 25 mM ethanolamine hydrochloride or with 25 mM ammonium as a control. The biomass yield was determined after 7 days of incubation. The *glnA4* mutant was not able to grow in the culture supplemented with ethanolamine. However, in defined Evans medium supplemented with ammonium, both the *glnA4* mutant and the parental strain grew well (Fig. 1). The parental strain formed pellets composed of a long and branched mycelium independently of the nitrogen source (ethanolamine or ammonium) added to the medium, whereas the *glnA4* mutant revealed no mycelial growth in the culture supplemented with ethanolamine (Fig. 4). Interestingly, only swollen and sporadic germinating spores were present in the *glnA4* mutant culture (Fig. 4C).

**FIG 5:**
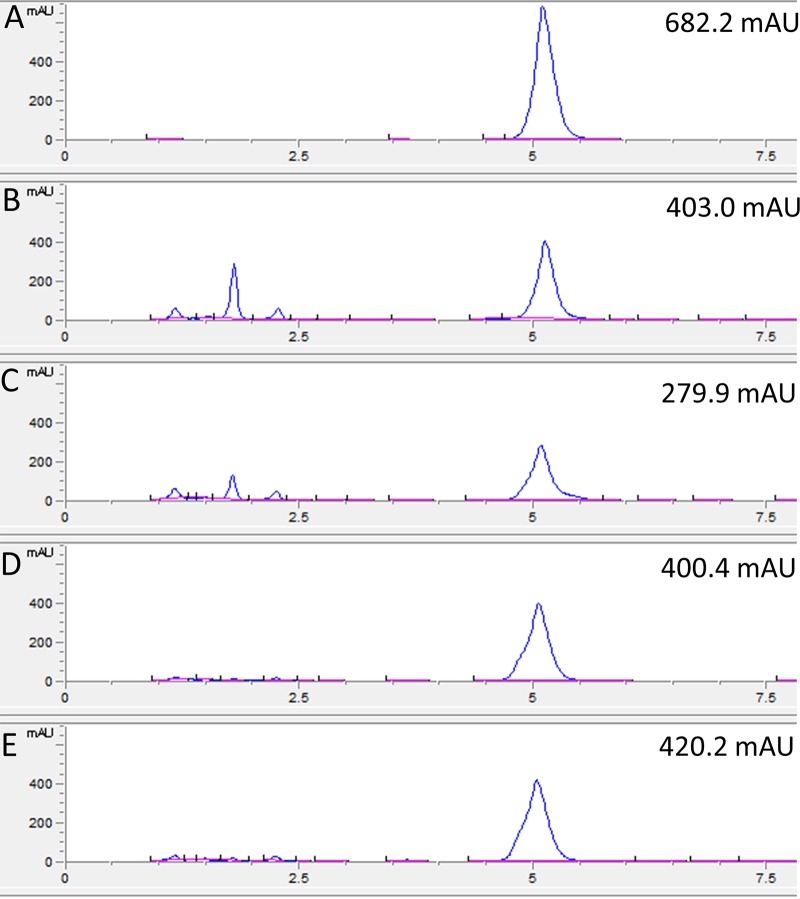
HPLC-based detection of ethanolamine in the supernatant from the 24-h and 96-h cultures of the M145 parental strain and *ΔglnA4* mutant. Both strains were grown in defined Evans medium supplemented with ethanolamine (25 mM) as the sole *N* source. The HPLC chromatogram data are presented as follows: A, ethanolamine standard; B and C, detection of ethanolamine remaining in the supernatant of the M145 culture after 24 h (B) and 96 h (C) of growth; D and E, detection of ethanolamine remaining in the supernatant of the Δ*glnA4* mutant culture after 24 h (D) and 96 h (E). The results indicate that only the S. coelicolor M145 parental strain was able to utilize more than one-third of ethanolamine from the medium after 96 h of incubation. mAU, milli-absorbance units.

To verify whether the *glnA4* mutant was able to utilize ethanolamine in the presence of other nitrogen sources, both the mutant and parental strains were cultivated in complex medium (yeast extract-malt extract [YEME]–Trypticase soy broth [TSB] [YEME-TSB] [33]) supplemented with 25 mM ethanolamine. Although the mutant was able to grow well in YEME-TSB supplemented with ethanolamine, its growth was inhibited compared to that of the parental strain. To confirm that the inhibition might have been a consequence of impaired ethanolamine utilization, the level of ethanolamine remaining in the culture medium after 24 h and 96 h of cultivation was determined using high-performance liquid chromatography (HPLC). Extracellular ethanolamine levels measured in cultures cultivated for 4 days with ethanolamine (25 mM) demonstrated significant differences. The level of ethanolamine determined in the supernatant from the *glnA4* mutant culture remained high during the entire incubation time, which was not the case for the level of ethanolamine in the supernatant from the parental strain culture (Fig. 5). These data indicated that the *glnA4* mutant was not able to utilize ethanolamine from the complex medium whereas the parental strain utilized more than half of the initial amount of ethanolamine. These results show that the absence of GlnA4 disallowed growth on ethanolamine, confirming a major role of GlnA4 in ethanolamine utilization in S. coelicolor.

### Expression of *glnA4* is induced under ethanolamine and starvation conditions.

**FIG 6:**
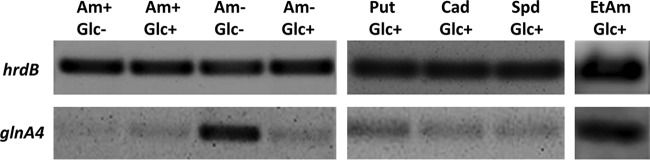
Transcriptional analysis of *glnA4* in the presence of ammonium, polyamines, and ethanolamine as sole nitrogen sources. Data represent results of reverse transcriptase PCR analysis of *glnA4* and *hrdB* (control) from S. coelicolor M145 cultivated in defined Evans medium with 5 mM (Am-) or 50 mM (Am+) concentrations of ammonium chloride, polyamines (Put, putrescine; Cad, cadaverine; Spd, spermidine, 25 mM each) or ethanolamine (EtAm; 25 mM) and glucose as the sole carbon source at a high concentration (Glc+; 25 g/liter) or a low concentration (Glc-; 2.5 g/liter). Total RNA was isolated from mycelium harvested after 24 h of cultivation in defined Evans medium.

The *glnA4* gene, annotated as “*glnA*-like,” encodes a protein that shows significant similarity to the glutamine synthetase (GlnA = GSI) and the gamma-glutamylpolyamine synthetase, GlnA3. The *glnA* gene is expressed constitutively (34, 35), and, as reported by Amin et al. (36), the expression of glutamine synthetase *glnA* is enhanced by low ammonium concentrations. In contrast, the expression of *glnA3* is induced by polyamines as well as under conditions of low *N* and *C* source concentrations (27). To test whether the expression pattern of *glnA4* might reflect some similarities to the *glnA* or *glnA3* expression patterns, a transcriptional analysis of *glnA4* was performed under various *N* conditions. For this purpose, S. coelicolor parental strain M145 was grown in complex S-medium for 4 days and was subsequently transferred into Evans medium supplemented with ethanolamine hydrochloride, ammonium chloride, or polyamines (putrescine, cadaverine, and spermidine) as the sole nitrogen source (25 mM each) and incubated further for 24 h at 30°C. Total RNA was isolated from S. coelicolor M145 and used to generate cDNA for reverse transcriptase PCR (RT-PCR) analysis with primers for *glnA4* (and *hrdB* as an internal control). Transcriptional analysis of *glnA4* revealed elevated expression levels of the *glnA4* gene in the presence of ethanolamine, a putative substrate of the encoded enzyme. *glnA4* gene expression was also enhanced under conditions of *N* and *C* starvation, namely, under conditions of small amounts of glucose (2.5 g/liter) and ammonium (5 mM) (Fig. 6). The level of *glnA4* gene expression was low in the presence of low ammonium concentrations and high glucose concentrations as well as under conditions of high polyamine concentrations and glucose proficiency, showing that the *glnA4* expression pattern is dissimilar to the previously reported expression patterns of *glnA* (36) and *glnA3* (27) under the same tested conditions.

### The expression of *glnA4* is regulated by the negative transcriptional regulator EpuRI.

Analysis of *glnA4* genomic localization revealed a gene encoding a putative regulatory protein (*SCO1614* [*epuRI*]). This regulatory gene is positioned upstream of *glnA4* in an orientation opposite that of *glnA4*. To verify whether this gene encodes a potential regulator of *glnA4*, a mutant with a knockout of the *epuRI* gene was generated. The growth of the *epuRI* mutant was monitored in liquid Evans medium supplemented with 25 mM ethanolamine hydrochloride or 25 mM ammonium chloride (control) as the sole nitrogen source. The biomass was determined after 3 to 7 days of incubation at 30°C and 180 rpm. The *epuRI* mutant demonstrated higher biomass accumulation in the culture with ethanolamine than parental S. coelicolor M145 strain. In the *epuRI* mutant culture grown on ethanolamine as the sole *N* source, the biomass doubled compared to the biomass of the parental strain grown under the same conditions. The *epuRI* mutant culture grown on ethanolamine as the sole *C* source revealed biomass three times as high as that of the parental strain grown under the same conditions. These results indicate that the deletion of *epuRI* positively influenced growth on ethanolamine (Fig. 1).

To study the effect of the *epuRI* deletion on the expression of *glnA4* in the presence of ammonium or ethanolamine, the cells of the M145 parental strain and the *epuRI* mutant were incubated in complex S-medium at 30°C for 4 days. Then, the pellets were washed twice with Evans medium to remove complex nitrogen sources and transferred into Evans medium with ammonium chloride (25 mM), ethanolamine hydrochloride (25 mM), or polyamines as the only nitrogen source and incubated for 24 h at 30°C. Total RNA was isolated and used to generate cDNA, which was then subjected to RT-PCR analysis using internal primers for *epuRI* and *hrdB.* Transcriptional analysis revealed strong expression of *glnA4* in the M145 parental strain in the presence of ethanolamine and in the *epuRI* deletion mutant in the presence of ethanolamine and ammonium (Fig. S8). This result indicates that *epuRI* encodes a repressor of *glnA4* because deletion of this regulator allows *glnA4* expression in the presence of ammonium.

10.1128/mBio.00326-19.8 FIG S8 Transcriptional analysis of *glnA4* in the presence of ethanolamine and ammonium as the sole nitrogen source. Reverse transcriptase PCR analysis of *glnA4* and *hrdB* (control) from S. coelicolor M145 and the *epuRI* mutant cultivated in defined Evans medium with ammonium chloride (25 mM) or ethanolamine (25 mM) was performed. Total RNA was isolated from mycelium harvested after 24 h of cultivation in defined Evans medium. Download FIG S8, TIF file, 1.0 MB. Copyright © 2019 Krysenko et al. 2019 Krysenko et al. This content is distributed under the terms of the Creative Commons Attribution 4.0 International license.

### GlnA4, a predicted gamma-glutamylethanolamide synthetase in S. coelicolor M145.

The GlnA4 protein, deduced from the *glnA4* DNA sequence, has been classified as a ligase that might form carbon-nitrogen bonds in an ATP-dependent manner, corresponding to class 6.3.1. This class includes 20 different subclasses of enzymes, e.g., glutamine synthetases (GS) (class 6.3.1.2), glutamate-ethylamine ligases (class 6.3.1.6), and glutamate-putrescine ligases (6.3.1.11). Analysis of the GlnA4 protein sequence by InterProScan predicted two enzymatic domains that resemble the *N*-terminal GS, beta-Grasp domain (IPR008147) and the *C*-terminal catalytic domain (IPR008146) of GS. Searching for functionally and structurally characterized GlnA4 homologs in the Protein Data Bank (PDB) revealed gamma-glutamylmonoamine/polyamine synthetase PauA7 (PA5508) from Pseudomonasaeruginosa and GSI*_St_* from *S.* Typhimurium as possible templates for a GlnA4 structural model. PauA7 is involved in monoamine/polyamine gamma-glutamylation in P. aeruginosa (37). The crystal structures of GS GSI*_St_* from *S.* Typhimurium (Protein Data Bank entry: 1FPY) and Pau7 (Protein Data Bank entry: 4HPP) were used as templates to generate a GlnA4 structural model. Superposition of the GS and PauA7 template structures and the GlnA4 model structure revealed conserved residues coordinating Mn^++^/Mg^++^ (N1 and N2), glutamate, and ATP. GSI*_St_* (as well as other eukaryotic and bacterial GS) and PauA7 require two divalent metal ions per subunit for activity. These ions have structural and catalytic roles. Comparison of the conserved catalytic residues with respect to the binding of divalent metal ions revealed six conserved residues, E156, E158, E219, E226, H274, and E359, in the GlnA4 model structure (corresponding to E131, E133, E180, E187, H236, and E322 in PauA7 and to E129, E131, E212, E220, H269, and E357 in GSI*_St_*).

Moreover, five conserved residues involved in the coordination of glutamate binding in GSI*_St_* and PauA7 were found in the GlnA4 model structure. These GlnA4 residues (E158, E269, G270, R325, and R361) correspond to E133, G234, N233, R290, and R334 in PauA7 as well as to E131, N264, G265, R321, and R359 in GSI*_St_*. Finally, two conserved residues (H276 and R348), both corresponding to H271 and R344 in GSI*_St_* as well as to H238 and R313 in PauA7, that are important for coordination of the beta-phosphate and alpha-phosphate groups of ADP were found in the GlnA4 model. This comparative *in silico* analysis demonstrated that GlnA4 possesses conserved residues for the binding of two metal ions, l-glutamate and ATP (Fig. S2 to S6).

10.1128/mBio.00326-19.2 FIG S2 Superposition of the GS*_St_* (1FPY) and PauA7 (4HPP) templates with the GlnA4 structural model and comparison of the Mg^++^/Mn^++^ binding pockets. Download FIG S2, TIF file, 0.4 MB. Copyright © 2019 Krysenko et al. 2019 Krysenko et al. This content is distributed under the terms of the Creative Commons Attribution 4.0 International license.

An essential element of the GSI*_St_* catalytic pocket is a loop termed “the E327 flap” (GSI*_St_*). The GSI active site is located between two subunits, and the E327 flap closes the catalytic site by interaction of E335 with D50 from an adjacent subunit within the same ring, shielding the gamma-glutamyl phosphate intermediate from hydrolysis. The second conserved residue, D50, is involved in the deprotonation of ammonium for attack on γ-glutamyl phosphate. Interestingly, these key acidic residues (E327 and D50) that are essential for the catalytic synthesis of glutamine in GSI*_St_* are not conserved in PauA7 or in GlnA4 (Fig. S2 to S6). In the PauA7 structure and the GlnA4 model, these loops are much larger, and instead of E327, a nonpolar F331 residue occupies the analogous position in the GlnA4 model structure (corresponding to W296 in PauA7). The conserved D50 residue that increases the affinity for ammonium binding in GSI*_St_* is replaced by G40 and D81/D69 (depending on the model) in PauA7 and GlnA4, respectively. Moreover, the conserved Y179 in GSI*_St_* that coordinates the ammonium binding pocket is substituted by A147 in PauA7 and by V189/D186 (depending on the model) in GlnA4, providing much more space for a substrate larger than ammonium, such as ethanolamine (Fig. S2 to S6). The significant overall similarity of GlnA4 to the gamma-glutamylmonoamine/polyamine synthetase PauA7, the lack of conserved residues for ammonium binding, and our observations strongly suggest that GlnA4 may function as a gamma-glutamylethanolamine synthetase catalyzing the first step of ethanolamine utilization.

### GlnA4 catalyzes the gamma-glutamylation of ethanolamine *in vitro*.

**FIG 7:**
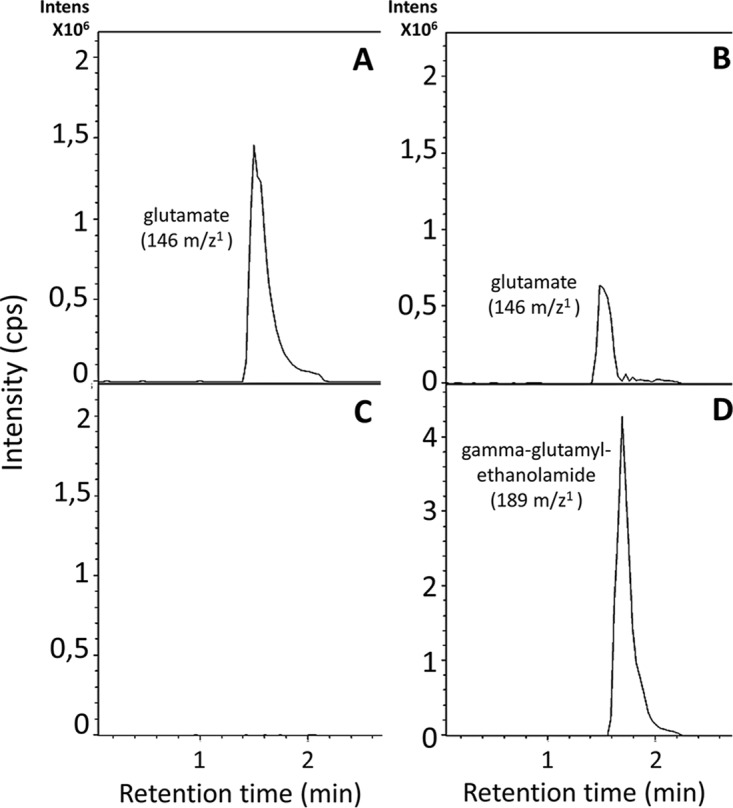
HPLC/MS analysis of the GlnA4-catalyzed reaction. Samples were taken after 0 (A and C) and 10 min (B and D) and analyzed in −MS mode. Extracted ion chromatograms for glutamate with a mass-to-charge ratio of 146 *m*/*z* (A and B) and for gamma-glutamylethanolamide with 189 *m*/*z* (D) are shown. Intens, intensity. (C) In the absence of the GlnA4 no product (gamma-glutamylethanolamide) could be detected.

To elucidate the function of GlnA4 and to show that GlnA4 is able to catalyze the predicted glutamylation reaction, we developed an HPLC/mass spectrometry (HPLC/MS)-based assay designed to detect a gamma-glutamylated product formed by GlnA4. GlnA4 was heterologously produced as a His-tagged protein in E. coli BL21(DE3) and purified by nickel ion affinity chromatography. Purified His-GlnA4 was used in an *in vitro* assay as described in Materials and Methods. The reaction conditions were as follows. A 10-μg volume of His-GlnA4 was incubated at 30°C in a reaction mixture composed of glutamate, ethanolamine, MgCl_2_, ATP, and HEPES buffer (pH 7.2). The product of the GlnA4-catalyzed reaction was detected using HPLC/MS analysis in positive MS mode. The results indicated that GlnA4 was able to use glutamate (mass-to-charge ratio of 146 *m*/*z*) (Fig. 7A and B) and ethanolamine as substrates in an ATP-dependent reaction, generating a product with a mass-to-charge ratio of 189 *m*/*z*, corresponding to the calculated mass of the predicted gamma-glutamylethanolamide (Fig. 7D) reaction product. The results of the HPLC/MS analysis confirm that GlnA4 is a functional gamma-glutamylethanolamide synthetase that uses glutamate and ethanolamine as substrates to generate gamma-glutamylethanolamide.

### Biochemical characterization of GlnA4.

To determine the kinetic parameters of GlnA4 and its substrate specificity, an adapted GS activity assay based on the inorganic phosphate (Pi) released from ATP was used (38). Pi released from ATP was measured using a colorimetric ammonium molybdate-based detection method. Phosphate that formed a complex with molybdate could be reduced in a strong acidic solution to form molybdenum blue. This change in color was measured by reading the absorption at 655 nm.

A total of 10 micrograms of purified His-GlnA4 was used, which was sufficient to produce between 50 and 100 nmol Pi in an incubation performed for 5 min at 30°C. For the quantification of the released inorganic phosphate, a calibration curve was prepared with potassium phosphate dibasic K_2_PO_4_ at concentrations of 20, 10, 5, 2.5, 1.25, 0.63, 0.31, 0.16, 0.08, 0.04, 0.02, and 0 mM. After the kinetic parameters of ATP were described, solution E was used to create an additional calibration curve with the same K_2_PO_4_ concentrations.

**FIG 8:**
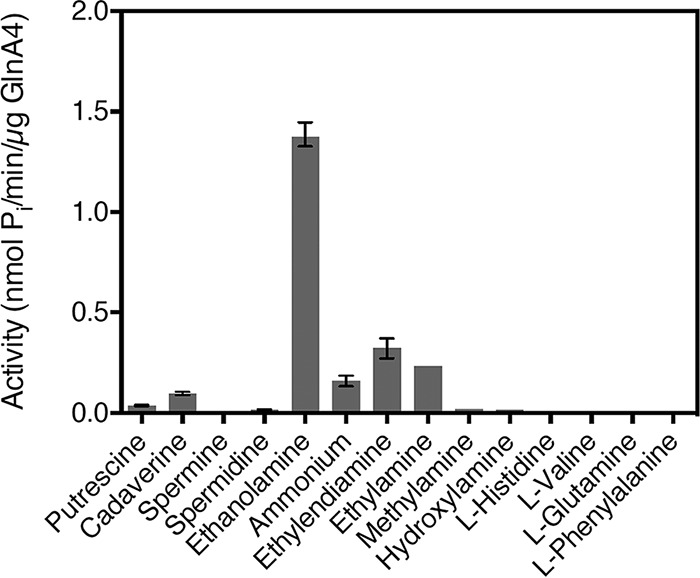
Effect of different amino group-containing compounds on the activity of GlnA4. All potential substrates of GlnA4 were present at a concentration of 50 mM. Mean values of *n* = 3 measurements are shown with standard deviations. GlnA4 is specific for ethanolamine.

To test the substrate specificity of GlnA4, this enzyme was incubated with different substrates at a concentration of 50 mM. To calculate the activity of GlnA4, the amount of released inorganic phosphate was quantified and expressed in nanomoles of Pi per minute per microgram of enzyme (Fig. 8; see also Fig. S7).

10.1128/mBio.00326-19.3 FIG S3 Superposition of the GS*_St_* (1FPY) and PauA7 (4HPP) templates with the GlnA4 structural model and comparison of the glutamate binding pockets. Download FIG S3, TIF file, 0.4 MB. Copyright © 2019 Krysenko et al. 2019 Krysenko et al. This content is distributed under the terms of the Creative Commons Attribution 4.0 International license.

10.1128/mBio.00326-19.4 FIG S4 Superposition of the GS*_St_* (1FPY) and PauA7 (4HPP) templates with the GlnA4 structural model and comparison of the ADP binding pockets. Download FIG S4, TIF file, 0.4 MB. Copyright © 2019 Krysenko et al. 2019 Krysenko et al. This content is distributed under the terms of the Creative Commons Attribution 4.0 International license.

10.1128/mBio.00326-19.5 FIG S5 Superposition of the GS*_St_* (1FPY) and PauA7 (4HPP) templates with the GlnA4 structural model and comparison of the ammonium binding pockets. Download FIG S5, TIF file, 0.4 MB. Copyright © 2019 Krysenko et al. 2019 Krysenko et al. This content is distributed under the terms of the Creative Commons Attribution 4.0 International license.

10.1128/mBio.00326-19.6 FIG S6 Superposition of the GS*_St_* (1FPY) and PauA7 (4HPP) templates with the GlnA4 structural model and comparison of the ammonium binding pockets. Download FIG S6, TIF file, 0.4 MB. Copyright © 2019 Krysenko et al. 2019 Krysenko et al. This content is distributed under the terms of the Creative Commons Attribution 4.0 International license.

10.1128/mBio.00326-19.7 FIG S7 Activity of GlnA4 with various concentrations of ethanolamine (A), glutamate (B), and ATP (C) and in the presence of MSO (D). A nonlinear regression (solid black line) was made using a least-squares fit of *n* = 3 data sets (white bullets) and assuming the Michaelis-Menten model. Download FIG S7, TIF file, 0.1 MB. Copyright © 2019 Krysenko et al. 2019 Krysenko et al. This content is distributed under the terms of the Creative Commons Attribution 4.0 International license.

The biochemical analysis revealed that GlnA4 accepts ethanolamine, glutamate, and ATP as substrates. It catalyzes the ATP-dependent synthesis of gamma-glutamylethanolamide from ethanolamine and glutamate. The formation of gamma-glutamylethanolamide and simultaneous depletion of glutamate were detected by HPLC/MS. Assuming the Michaelis-Menten model, the *K_m_* values were determined to be 0.47 mM for ethanolamine, 85.41 mM for glutamate, and 2.5 mM for ATP. The highest *V*_max_ value of 4.18 nmol Pi per min per 1 μg of enzyme was determined for glutamate, while *V*_max_ values of 1.48 and 0.76 nmol Pi per min per 1 μg of GlnA4 were determined for ethanolamine and ATP, respectively (Fig. S7).

Incubation of GlnA4 with the reaction mixture containing methionine sulfoximine (MSO; a potent inhibitor of glutamine synthetases) only slightly lowered the activity of GlnA4 (Fig. S7). The estimated *K_i_* value (11 mM) was approximately 10 to 5,000 times higher than the *K_i_* values reported for glutamine synthetases (39–42), indicating that MSO is not a potent inhibitor of GlnA4.

## DISCUSSION

*Streptomycetes* spp. have a vast metabolic potential and are able to assimilate nitrogen from a variety of minerals (e.g., ammonium, nitrate, and nitrite) and organic sources (e.g., urea, amino acids, peptides, amino sugars, and polyamines). However, a nitrogen source such as ethanolamine, which is also omnipresent in nature, has never been reported to be utilized by S. coelicolor. In this study, we demonstrated that Streptomyces coelicolor M145 can utilize ethanolamine as a sole carbon and nitrogen source. However, the slow growth of Streptomyces coelicolor M145 on medium with ethanolamine as the sole *N*/*C* source showed that ethanolamine was not a preferred *N*/*C* source for growth under the tested conditions.

Depending on conditions, extracellular ethanolamine can enter the cell through diffusion, carrier-mediated transport (19), or, alternatively, with the help of transport proteins, such as permease-like proteins EutH in E. coli and *S.* Typhimurium (19, 43) and Eat in *Actinobacteria* and most *Proteobacteria* (18). Screening of *Streptomyces* genomes revealed no strong homologs of EutH or Eat. However, genes annotated as ethanolamine/amino acid permeases with no similarity to EutH or Eat were found. Screening of the S. venezuelae, S. pristinaespiralis, S. griseus, S. albus, and S. scabies genomes revealed a gene encoding a putative ethanolamine/amino acid permease located in the gene cluster containing the *glnA4* gene. Introduction of one of the predicted ethanolamine permease genes (*spri_5940* from S. pristinaespiralis) into S. coelicolor positively influenced the growth of S. coelicolor-pRM*spri_5940* on defined medium supplemented with ethanolamine as the sole *N* source. It is likely that an ethanolamine permease-encoding gene was lost in the S. coelicolor genome during the evolution process; however, the rest of the ethanolamine utilization cluster was maintained, presumably to ensure intracellular ethanolamine recycling. S. coelicolor has a plethora of sugar, organic anion, and amino acid uptake systems of very specific types (total, 658 transporter proteins) (44). The possibility of ethanolamine uptake occurring via an alternative transporter with low affinity cannot be excluded. This kind of transporter might belong to the amino acid-polyamine-organocation (APC) superfamily consisting of 17 predicted members (44).

Intracellular ethanolamine content can increase as a result of glycerophospholipid polar head recycling, as shown in Mycobacterium tuberculosis (45). Phosphodiesterase (Rv3842) breaks down lipid polar heads of glycerophosphoethanolamine, glycerophosphoglycerol, or glycerophospho-1-d-myo-inositol, releasing glycerol phosphate (45). The released by-product of glycerophosphoethanolamine cleavage is ethanolamine, which can be further metabolized as a *C* and *N* source. The ethanolamine level in the cell must be strictly controlled due to its potential toxic effect when present in excess. Intracellular ethanolamine accumulation might lead to alkalization of the cellular milieu, unspecific binding to negatively charged molecules such as DNA and RNA, compromised cellular homeostasis, and, ultimately, cell death. Since the expression of *glnA4* is not only induced by ethanolamine but also under the control of nutrient limitation, it can be concluded that GlnA4 may play a supporting role during starvation. Under such conditions, cellular membranes likely undergo degradation, increasing extra and intracellular ethanolamine concentrations.

The parental strain S. coelicolor M145 is able to grow on ethanolamine as a sole *N*/*C* source. Relating to our studies, this ability is due to the catalytic activity of GlnA4. The deletion of *glnA4* resulted in a growth defect for S. coelicolor M145 in liquid medium, strongly impaired growth on solid medium, and affected cell morphology in the presence of ethanolamine. *In vitro* analysis of the enzymatic activity of GlnA4 revealed that GlnA4 functions as a gamma-glutamylethanolamide synthetase. Our study showed that GlnA4 is specific to ethanolamine and catalyzes its glutamylation using glutamate and ATP. Thus, GlnA4 can be classified in a large class of ligases that can form a carbon-nitrogen bond in an ATP-dependent manner (class 6.3). Although GlnA4 resembles features of glutamine synthetases (GSs), it is not able to catalyze glutamine biosynthesis. Currently, there are few examples described in the scientific literature in reports of glutamine synthetase-like enzymes that catalyze the glutamylation of various *N* compounds but lack glutamine synthetase activity. For instance, Kurihara et al. (46) described gamma-glutamylputrescine synthetase PuuA, which catalyzes ATP-dependent gamma-glutamylation of putrescine in E. coli. Krysenko et al. (27) demonstrated that gamma-glutamylpolyamine synthetase GlnA3 catalyzes the glutamylation of putrescine, cadaverine, spermidine, and spermine in S. coelicolor. Furthermore, seven gamma-glutamylpolyamine/monoamine/aromatic amine synthetases (PauA1 to PauA7) have been described in Pseudomonas aeruginosa PAO1 (37, 47). PauA7 is the only structurally characterized glutamine-like enzyme with the ability to glutamylate aromatic monoamines. PauA7 exhibits a structure different from that seen with the common glutamine synthetase archetype, forming only a single hexameric ring instead of two (37). Whether GlnA4 forms one or two rings and how its catalytic pocket differs from that of glutamine synthetases are under investigation.

The glutamylation reaction is common in nature. For example, gamma-glutamyltransferases can catalyze the transfer of gamma-glutamyl groups from glutathione to various amino acids or peptides (48); polyglutamylases catalyze the glutamylation of proteins (49); and gamma-glutamylpolyamine/monoamine or aromatic amine synthetases catalyze the glutamylation of various amines (27, 37, 46, 47). Furthermore, gamma-glutamyl compounds, e.g., peptidoglycan, glutathione (gamma-glutamylcysteinylglycine), poly-gamma-glutamic acid, glutamylated proteins, gamma glutamylpolyamines/monoamines, and gamma-glutamyltaurine, are not rare in nature (50). The glutamylation of compounds was shown to positively influence their chemical properties, for instance, to increase solubility, change the taste or aroma, increase stability in solution or half-life in blood serum, and reduce toxicity (50). Therefore, the biotechnological application of gamma-glutamylating enzymes is of high interest. For example, theanine (l-gamma-glutamylethylamide), which is a major aromatic “umami” component of tea and which has thus far been extracted from tea plants, might be able to be biotechnologically synthesized using only enzymatic properties of GlnA4. Altering the binding affinity of GlnA4 through protein engineering such that ethylamine would be preferred as a substrate instead of ethanolamine would lead to the effective and sustainable synthesis of theanine.

The *glnA4* gene is annotated as a glutamine synthetase-like gene and is one of the three *glnA*-like genes in S. coelicolor. The glutamine synthetase-like genes are also found multiple times in the phylum *Actinobacteria*, including the human pathogen Mycobacterium tuberculosis (28). Sequence analysis showed that *glnA1-* and *glnA2*-encoded glutamine synthetase sequences were inherited from an *Actinobacteria* ancestor whereas *glnA4* was sequentially acquired during *Actinobacteria* speciation. In particular, in *Mycobacteria*, a reductive evolution of *glnA4* can be observed, while *glnA1* and *glnA2* seem to be more extensively conserved. However, to date, there has been no description of a glutamine-synthetase-like enzyme in *Actinobacteria*, making the characterization of GlnA4 as a gamma-glutamylethanolamide synthetase the first such characterization.

In this work, novel insights into the regulatory network of ethanolamine utilization genes, including *glnA4*, were developed. The issue of the regulation of *glnA4* has remained unsolved thus far, since it was shown that GlnR and GlnRII, the global transcriptional regulators of nitrogen assimilation genes, do not bind the promoter regions of *glnA*-like genes (34, 35, 51). Additionally, a genome-wide analysis of GlnR-regulated genes in M. smegmatis demonstrated that a homolog of GlnA4 was not regulated by the global nitrogen-response regulator GlnR (52). The *epuRI* (*sco1614*) gene is annotated as a putative transcriptional regulator in the genome of S. coelicolor (53) and is localized upstream of *glnA4*. Our studies suggest that EpuRI can act as a negative transcriptional regulator of *glnA4*.

The ability of S. coelicolor to utilize ethanolamine as an *N* and *C* source with the help of GlnA4 allowed the prediction of the ethanolamine utilization pathway. S. coelicolor does not possess a canonical ethanolamine utilization pathway that involves *eut* genes. Additionally, no homologs of *eut* genes of E. coli or *S.* Typhimurium were found in the S. coelicolor genome. However, the ethanolamine utilization pathway of S. coelicolor may look similar to the previously unknown ethanolamine utilization pathway in Chromohalobacter salexigens, which was predicted in an *in silico* approach, using the EFI-GNT Web tool, and is based on glutamylation (54). In S. coelicolor, the gene encoding GlnA4 is localized near four genes that may be involved in the utilization of ethanolamine. After gamma-glutamylethanolamide is generated by GlnA4, it may be oxidized to a gamma-glutamylacetaldehyde by a predicted dehydrogenase (SCO1611). Subsequently, gamma-glutamylacetaldehyde might be converted to gamma-glutamylglycine by a predicted gamma-glutamylaldehyde dehydrogenase (SCO1612). The gamma-glutamylglycine may then be hydrolyzed to glycine and l-glutamate by a member of the formylglutamate amidohydrolase family (e.g., by a predicted gamma-glutamylglycine amidohydrolase [SCO1615]) (see Fig. S9 in the supplemental material). Alternative pathways which do not require a metabolosome and do not result in the production of toxic by-products (such as acetaldehyde) were also reported in *Mycobacterium* spp. and C.salexigens (7, 8, 54–56). For instance, in *Mycobacterium*, ethanolamine can be converted via glycoaldehyde and glyoxalate intermediates to glycine, which can be subsequently converted to serine and alanine (7, 55).

10.1128/mBio.00326-19.9 FIG S9 Combined model of bacterial ethanolamine metabolism (7, 20, 54–56). ADP, adenosine diphosphate; ATP, adenosine triphosphate; Pi, phosphate; CoASH, coenzyme A; Glu, glutamate; NAD^+^/NADH, nicotinamide adenine dinucleotide. Dashed arrows represent transport by diffusion. Download FIG S9, TIF file, 1.7 MB. Copyright © 2019 Krysenko et al. 2019 Krysenko et al. This content is distributed under the terms of the Creative Commons Attribution 4.0 International license.

In this work, we demonstrated the function of GlnA4 as a gamma-glutamylethanolamide synthetase and its importance for ethanolamine catabolism in S. coelicolor. A better understanding of ethanolamine utilization in *Streptomyces* is of fundamental importance since this pathway allows the survival of *Actinobacteria* spp. living in diverse habitats. Our work represents the first attempt to reveal the ethanolamine utilization pathway in the actinobacterial model organism S. coelicolor through the characterization of GlnA4, a key enzyme that is absolutely required for survival under conditions of high ethanolamine concentrations. The ethanolamine utilization pathway is of great benefit for *Streptomyces* spp. that live under constantly unstable nutritional conditions and are forced to compete with other soil microorganisms and plants for the nitrogen available in the soil.

## MATERIALS AND METHODS

### Strains and growth conditions.

The S. coelicolor M145 parental strain and all mutants (Table 1) were incubated for 4 to 5 days at 30°C on defined Evans agar base (modified from the version described previously by Evans et al. [57]) supplemented with the following different nitrogen sources: 50 mM ammonium chloride, l-glutamine, sodium nitrate, monosodium glutamate, urea, and ethanolamine hydrochloride (in appropriate concentrations). For growth experiments in liquid culture, complex S-medium (58), YEME-TSB (33), or chemically defined Evans medium (57) was used. If appropriate, media were supplemented with ethanolamine hydrochloride or ammonium chloride as a sole nitrogen source. Strains were cultivated for 4 to 5 days at 30°C on a rotary shaker (180 rpm). Genetic manipulation of S. coelicolor M145 was performed as described by Kieser et al. (59) and Gust et al. (60). For genomic DNA preparation, S. coelicolor M145 was grown for 4 days in S-medium, and DNA was isolated by the use of a NucleoSpin tissue kit (Macherey-Nagel, Düren, Germany).

**TABLE 1:** Strains and plasmids used in this work

Strain or plasmid	Genotype or description^*a*^	Source or reference
E. coli XL1-Blue	*recA1 endA1 gyrA96 thi-1 hsdR17 supE44 relA1 lac* [F0 *proAB lacI*^q^*Z1*M15 Tn*10* TetR]	63
E. coli S17-1	*recA pro mod*^+^ *res*^−^ *tra* genes from plasmid RP4 integrated in the chromosome	64
S. coelicolor M145	Streptomyces coelicolor A3(2) without native plasmids: *spc1*^−^ and *spc2*^−^	59
S. pristinaespiralis ATCC 25486	Streptomyces pristinaespiralis strain ATCC 25486	Fischbach et al.^*b*^
S. coelicolor M145 *ΔglnA4*	*glnA4* mutant strain of S. coelicolor M145; *glnA4* replaced by an *aac*(*3*)*IV* cassette; Apr^r^	This work
S. coelicolor M145 *ΔglnA4*pRM4*glnA4*	Complemented *glnA4* mutant strain of S. coelicolor M145; Apr^r^ and Km^r^	This work
S. coelicolor M145 *ΔepuRI*	*epuRI* mutant strain of S. coelicolor M145; *epuRI* replaced by an *aac*(*3*)*IV* cassette; Apr^r^	This work
S. coelicolor M145 pRM4*spri_5940*	Heterologous overexpression strain of S. coelicolor M145 with pRM4*spri_5940*; Apr^r^	This work
pRM4	pSET152ermEp* with artificial RBS; Apr^r^	65
pIJ10700	pBluescript II KS(C) containing *hyg-oriT* cassette; Hyg^r^	60

a Apr^r^, apramycin resistance; Hyg^r^, hygromycin resistance; Km^r^, kanamycin resistance; RBS, ribosome binding site.

b Sequence submitted to the EMBL/GenBank/DDBJ databases by M. Fischbach, P. Godfrey, D. Ward, S. Young, Q. Zeng, M. Koehrsen, 21 October 2009.

### Construction of the *ΔglnA4* mutant.

The *glnA4* gene was replaced by the apramycin resistance gene using plasmid pK18 carrying homologous regions of *glnA4*. The construct was confirmed by sequencing and subsequently introduced into S. coelicolor M145 by conjugation using the E. coli S17 strain. Conjugants with an apramycin-resistant phenotype were then selected. The correct integration of pRM4*sco1613* was confirmed by PCR and sequencing.

### Construction of the M145pRM4*spri_5940* overexpression mutant.

To enable the heterologous expression of ethanolamine permease from S. pristinaespiralis in S. coelicolor M145, the *spri_5940* gene without its native promoter was amplified by PCR using SPRI_5940_F and SPRI_5940_R primers (Table 2) and cloned into the multiple-cloning site of the pRM4 plasmid between the NdeI and HindIII restriction sites. The construct was confirmed by sequencing and subsequently introduced into S. coelicolor M145 by conjugation using the E. coli S17 strain. Conjugants with an apramycin-resistant phenotype were then selected. The correct integration of pRM4-*spri_5940* was confirmed by PCR and sequencing.

**TABLE 2:** Oligonucleotides used in this work

Oligonucleotide	Sequence (5′–3′)	Source or reference
SPRI_5940_F	CGCCCATATGGCTGAAGGCACCACATC	This work
SPRI_5940_R	TCCAAGCTTTCACTTCCGTTCCAGTTC	This work
pRM_GenF	CTGCAAGGCGATTAAGTTGG	This work
pRM_GenR	TTATGCTTCCGGCTCGTATG	This work
spri_5940F_RT	GCTGTAGAAGGCGAAGTAGG	This work
spri_5940R_RT	CGATCAGCTACGCGCTGATG	This work
hrdB-qrt1	TGACCAGATTCCGGCCACTC	This work
hrdB-qrt2	CTTCGCTGCGACGCTCTTTC	This work
rt-glnA4-fw	CGCCTGGGACGCGAACTAC	66
rt-glnA4-rev	CTGGGCGGCGATCTCCTTG	66
RT1614N4F	GGACACGATGCTGCACCTGA	This work
RT1614N4R	TGCAGTCCAGCAGGTCGTTC	This work
rt_sco1612up	TCACGCTCCTCGAACCACTC	This work
rt_sco1612dw	CGTCGGCGAAGACGATGTTG	This work
RT5977F	TTCCAGGACGGCAACCTCAC	This work
RT5977R	AAGACCACGCCGACGATCAG	This work
RT6014F	GTCAAGAGCGCCAACTAC	This work
RT6014R	TACAGGAGGGTGCAGATG	This work
SCO1614_f	AAAAAGCTTCAGCACCTGGAACAGGTCCT	This work
SCO1614_r	AAAATCTAGAACCGGCTCATCGTCGTCACT	This work
1614_BamHI	AAAGGATCC TCAGTGGTGGTGGTGGTGG	This work
1614-NdeI	AAAAAACATATGACGGACCGGCTGGCGC	This work
SCO1614-XhoI	AAAACTCGAGCGTCAGAAACCCGCGCAGCA	This work
SCO1614-NcoI	AAAACCATGGGCACGGACCGGCTGGCGCCG	This work

### Construction of the *ΔglnA4* complementation mutant.

For complementation of the mutant, the *glnA4* gene with its native promoter was amplified by PCR using *glnA4_*XbaI and *glnA4_*EcoRI primers (Table 2) and cloned into the multiple-cloning site of the pRM4 plasmid between the XbaI and EcoRI restriction sites in the opposite orientation to the promoter P*emrE*, which is located on the plasmid. Subsequently, the kanamycin resistance cassette was amplified by PCR using the pK18 plasmid as a template and primers *aphIIupperEcoRI* and *aphIIlowerHindIII* and introduced into the multiple-cloning site of recombinant plasmid pRM4-*glnA4C* between the EcoRI and HindIII restriction sites. The correct construct was confirmed by sequencing and subsequently introduced into the *ΔglnA4* mutant by conjugation using the E. coli S17 strain. Clones with a kanamycin-resistant and apramycin-resistant phenotype were then selected. The correct integration of pRM4-*glnA4* was confirmed by PCR and sequencing.

### Construction of the *ΔepuRI* mutant.

To generate the in-frame deletion of the *epuRI* (*sco1614*) gene, the REDIRECT gene replacement procedure (60) was employed. The *epuRI* gene was replaced by the apramycin resistance gene. The construct was confirmed by sequencing and subsequently introduced into S. coelicolor M145 by conjugation using the E. coli S17 strain. Conjugants were then selected on a resistant phenotype against apramycin. The correct integration of pRM4-*epuRI* was confirmed by PCR and sequencing.

### Analysis of gene expression by reverse transcriptase PCR.

For transcriptional analysis experiments, S. coelicolor M145, the *glnA4* mutant, and the *epuRI* mutant were cultured in S-medium. After 4 days of incubation, cells were washed twice with defined Evans medium and cultivated for 24 h in defined Evans medium supplemented with 25 mM ammonium chloride, 25 mM polyamine (cadaverine, spermidine, or putrescine), or 25 mM ethanolamine. RNA was isolated with an RNeasy kit (Qiagen, Venlo, The Netherlands) and treated twice with DNase (Thermo Fisher Scientific, Waltham, MA, USA). First, an on-column digestion was carried out for 30 min at 24°C, and then RNA samples were treated with DNase for 1.5 h at 37°C. RNA concentrations and quality were checked using a NanoDrop ND-1000 spectrophotometer (Thermo Fisher Scientific, Waltham, MA, USA). For the generation of cDNA from 3 μg RNA, random nonamer primers (Sigma-Aldrich Chemie GmbH, Munich, Germany) and reverse transcriptase and cofactors (Thermo Fisher Scientific, Waltham, USA) were used. PCRs were performed with the primers listed in Table 2. The PCR conditions were 95°C for 5 min; 35 cycles of 95°C for 15 s, 55 to 60°C for 30 s, and 72°C for 30 s; and 72°C for 10 min. As a positive control, cDNA was amplified from the major constitutively produced vegetative sigma factor (*hrdB*) transcript. Negative controls were carried out by using total RNA as a template for each RT-PCR to exclude DNA contamination. RT-PCRs were performed three times using RNA isolated from three different cultures.

### Cloning, expression, and production and purification of His-GlnA4.

The gene encoding GlnA4 (SCO1613) was amplified by PCR from S. coelicolor M145 genomic DNA and ligated into expression vector pJOE2775 between the NdeI and HindIII restriction sites under the control of rhamnose-inducible promoter P*rha*. His-GlnA4 was produced in E. coli strain BL21(DE3) grown in LB medium with ampicillin. Cells were grown initially at 37°C overnight, shaken at 180 rpm on a rotary shaker, transferred into a main culture, and incubated until the culture density reached an optical density of ∼0.5. Subsequently, induction with 20% rhamnose and incubation for an additional 5 h were performed. The cells were harvested and stored at −20°C until needed. His-GlnA4 was purified by nickel ion affinity chromatography essentially as directed by the resin manufacturer (GE Healthcare, Munich, Germany). Purified His-GlnA4 was dialyzed against 20 mM Tris and 100 mM NaCl (pH 8) and immediately used for further analysis.

### GlnA4 *in vitro* assay and HPLC/MS detection of the glutamylated product.

The HPLC/MS method was used to evaluate ethanolamine and glutamate as GlnA4 substrates as well as the generated gamma-glutamylated ethanolamine as a product. Reaction mixtures typically contained 20 mM HEPES (pH 7.2), 150 mM glutamate sodium monohydrate, 150 mM ethanolamine hydrochloride, 20 mM MgCl_2_ × 6H_2_O, and 10 mM ATP mixed with 10 μg of purified His-GlnA4 (or, as a control, in the absence of GlnA4) and were incubated at 30°C for 10, 20, and 30 min and 1 h. The reaction was stopped by incubation of the reaction mixture at 100°C for 5 min. HPLC/ESI-MS analysis of the glutamylated product generated by GlnA4 was performed on an Agilent 1200 HPLC series system using a Reprosil 120 C_18_ AQ column (5-μm pore size, 200 by 2 mm) fitted with a precolumn (Dr. Maisch GmbH, Ammerbuch, Germany) (10 by 2 mm) coupled to an Agilent LC/MSD XCT 6330 Ultra Trap system (Agilent, Waldbronn, Germany). Analysis was carried out using 0.1% formic acid as solvent A and acetonitrile with 0.06% formic acid as solvent B at a flow rate of 0.4 ml min^−1^. The gradient was as follows: *t*_0_ to *t*_5_ = 0% B and *t*_20_ = 40% B (where “*t*” represents time in minutes). The injection volume was 2.5 μl, and the column temperature was 40°C. ESI ionization was performed in positive mode with a capillary voltage of 3.5 kV and a drying gas temperature of 350°C.

### Enzymatic activity assay.

To elucidate the enzymatic reaction catalyzed by GlnA4, a modified GS activity assay previously described by Gawronski and Benson (38) was adopted. Solutions A, B, C, and F and a reaction mixture containing enough protein that 35 to 50 mM Pi would be produced in 5 min were prepared. The pH was adjusted, and a 95-μl volume was loaded into PCR strips for each reaction. Solution D (or solution E, when kinetic parameters of ATP were to be determined) was prepared, and the reaction was initiated by adding 5 μl substrate to the reaction mixture. Additionally, deionized H_2_O (blank) and a phosphate standard (ranging from 0 to 20 mM) were included. The reaction mixture was incubated at 30°C for 5 min in a thermocycler. Meanwhile, 150 μl of solution D (or solution C) for each reaction was loaded into wells of a 96-well plate, and 50 μl of the reaction mixture was transferred to previously prepared solution D (or solution C) in the 96-well plate. The solutions were mixed well and incubated for 5 min at room temperature. The low pH of the solution terminated the enzymatic reaction, while 150 μl of solution F was added to stop color development. The final reaction mixture was incubated for 15 min at room temperature, and the absorbance was measured at 655 nm using a microplate reader. Raw absorbance readings were put into Excel (Microsoft), and the least-squares fit to the Michaelis-Menten equation was calculated with Prism 6 (GraphPad Software, Inc.).

To test the inhibitory effect of methionine sulfoximine on the enzymatic activity of GlnA4, a reaction mixture containing 20 mM HEPES (pH 7.2), 25 mM glutamate sodium monohydrate, 50 mM ethanolamine hydrochloride, 20 mM MgCl_2_ × 6H_2_O, 2,5 mM ATP, 2 μM purified His-GlnA4, and MSO at 0.5 to 5.000 μM was used. Raw absorbance readings were normalized to GlnA4 activity in the absence of MSO, and the 50% inhibitory concentration (IC_50_) value was determined using 4PL-fit implemented in Prism 6. From this value, the *K_i_* value was calculated using an equation previously described by Cheng and Prusoff (61).

### Estimation of the extracellular ethanolamine level using HPLC.

Extracellular ethanolamine levels were measured by the reverse-phase high-performance liquid chromatography (RT-HPLC) method previously described by Potter and Paton (62), which was optimized for S. coelicolor. The cells were centrifuged (6,000 × *g*, 10 min, 10°C), and the supernatant was transferred into new tubes. For protein precipitation, trichloroacetic acid (TCA) was added to reach a final concentration of 10%, and the mixture was incubated for 5 min on ice. Then, the mixture was cleared by centrifugation (13,000 × *g*, 10 min at 4°C), and the pH was optimized using HCl or NaOH. The samples were stored at −20°C until analysis. Ethanolamine was derivatized using precolumn derivatization with *ortho*-phthalaldehyde (OPA)/mercaptoethanol (MCE) (Dr. Maisch GmbH, Ammerbuch, Germany). OPA-derivatized ethanolamine was separated on a Reprosil OPA column (150 mm by 4.0 mm, 3 mm) fitted with a precolumn (Dr. Maisch GmbH, Ammerbuch, Germany) (10 mm by 4 mm, same stationary phase) using an HP1090 liquid chromatograph equipped with a diode-array detector, a thermostated autosampler, and an HP Kayak XM 600 ChemStation (Agilent, Waldbronn, Germany). UV detection was performed at 340 nm. The following gradient was used at a flow rate of 1.1 ml/min with solvent A (25 mM sodium phosphate buffer [pH 7.2] containing 0.75% tetrahydrofuran) and solvent B (25 mM sodium phosphate buffer [pH 7.2] [50%], methanol [35%], acetonitrile [15%] by volume): *t*_0_ = 35% B, *t*_16_ = 100% B, *t*_22_ = 100% B, and *t*_23_ = 35% B (where “*t*” represents time in minutes). Ethanolamine hydrochloride was purchased from Sigma and used as a standard. Solutions of standards were dissolved in distilled water.

### Survival assay.

The survival ability of S. coelicolor M145 and the *ΔglnA4* mutant in the presence of ethanolamine was examined by estimation of the dry weight biomass after 72 and 144 h of cultivation in the YEME-TSB (1:1)-rich complex medium. The M145 parental strain and *ΔglnA4* mutants were inoculated into YEME-TSB supplemented with ethanolamine (25 mM) and incubated for 3 days at 30°C. As a control, strain M145 and the *ΔglnA4* mutant were also cultivated in YEME-TSB without ethanolamine supplementation but with ammonium. Samples (1 ml) were harvested by centrifugation (16,200 × *g*, 15 min, 4°C). The supernatant was removed, and pellets were dried for 24 h at 100°C. Means of values from three technical replicates were used.
